# Improvement of Adeno-Associated Virus (AAV)-Based Technologies by Cell-Penetrating Penta-Peptides (CPP5s)

**DOI:** 10.3390/pharmaceutics18030395

**Published:** 2026-03-22

**Authors:** Charles W. Guo, Anastasia Diener, Shigemi Matsuyama

**Affiliations:** 1 Department of Ophthalmology and Visual Sciences, School of Medicine, Case Western Reserve University, Cleveland, OH 44106, USA; cwg40@case.edu (C.W.G.); aed134@case.edu (A.D.); 2 Case Comprehensive Cancer Center, Cleveland, OH 44106, USA

**Keywords:** Cell-Penetrating Peptide (CPP), Cell-Penetrating Penta-peptide (CPP5), adenovirus vector (AAV), nanoparticle, gene delivery, blood–brain barrier, blood retinal barrier, Ku70, Bax

## Abstract

Adeno-associated viruses (AAVs) are a promising gene therapy technology, but major technical challenges remain. One problem is that commonly used AAVs have a low efficiency in penetrating the blood–brain barrier (BBB) and the blood–retina barrier (BRB). Consequently, gene delivery to the nervous system has limitations. Another problem is that AAVs induce immune reactions that cause serious side effects. To avoid immune reactions, the AAV dose must be reduced to lower levels that may result in insufficient gene delivery. Researchers have been modifying viral capsid protein sequences and searching for effective peptide sequences to solve these problems. As a result, Cell-Penetrating Penta-Peptides (CPP5s) have been shown to be effective in improving the BBB/BRB penetration of AAVs and suppressing immune reactions against AAVs. CPP5s were originally developed from peptide sequences of the Bax (a pro-apoptotic protein) binding domain of Ku70 (a DNA repair protein) and from negative control cell-penetrating peptides without Bax-binding activity. This article will discuss the background science of CPP5s and future directions of CPP5s for AAV-mediated gene delivery to the nervous system as well as other organs.

## 1. Introduction

Cell-Penetrating Penta-Peptides (CPP5s) are a group of penta-peptides with the ability to deliver cargo molecules (e.g., proteins) into a cell, which were developed by the Matsuyama laboratory [1,2,3,4]. Recently, CPP5s have been rediscovered as useful tools to improve the efficacy and safety of Adeno-associated virus (AAV)-based gene therapy technology. This manuscript will summarize the background science of CPP5s and review recent publications utilizing CPP5s for the improvement of AAV gene therapy-related technology.

AAVs have become an FDA-approved delivery method for human gene therapy medicines [5,6,7,8]. These vehicles are increasingly chosen for their low pathogenicity, ability to transduce non-dividing cells, and reduced immune responses [5,9,10]. Although AAVs have shown effective gene transfers in several organs such as the liver, skeletal muscle, and bone marrow, they are limited for neurological disorders due to the difficulties of viral delivery across the blood–brain barrier (BBB) and the blood–retina barrier (BRB) [11]. While certain serotypes such as AAV9 have been shown to cross the BBB from intravascular administration, its transduction efficiency remains lower compared to other tissues [12]. To improve efficacy, higher doses of AAVs are needed, but this approach increases toxicity by eliciting greater host immune response [13,14]. Notably, AAV gene therapy has been linked to 11 patient fatalities since 2023, leading to concerns for patient safety [15,16,17]. Current AAV research aims to improve tissue specificity, transduction efficiency, genome expression, and reduced host immune responses [18].

To improve the BBB penetration, researchers have been investigating the effects of the modification of the capsid surface proteins essential for the virus–host cell interaction [19,20,21,22,23]. As one of these attempts, a group of researchers examined Cell-Penetrating Peptides (CPPs) to modify the AAV’s surface proteins [24,25,26,27]. CPPs are a group of short peptides ranging from 5 amino acids to over 40 amino acids that have been shown to have an activity to deliver cargo molecules (proteins (e.g., immunoglobulin or Green Fluorescence Protein (GFP)), small molecules, and nucleic acids (DNA and RNA)) [28]. For their cargo delivery activity, CPPs are directly attached to the cargo (especially in the case of protein) or attached to the delivery-packaging tool such as liposomes [29]. These CPPs then facilitate cellular uptake by overcoming the cell membrane barrier [30,31,32,33,34], though the precise molecular mechanism of cell entry and cargo delivery has not been fully understood [4,35]. Previous studies suggest that host cells continuously internalize and recycle their cell surface area, facilitating the entry of CPPs through endocytosis pathways [36,37]. Although CPPs can generally facilitate the penetration of plasma membrane, the efficacy of cargo delivery is influenced by heterogeneity of the cell membrane of each cell types [38].

By engineering AAV viral capsids with CPPs, it has been expected to improve the poor tissue selectivity and low transduction efficiency of AAVs [39]. For example, AAVv128 is a synthetic AAV8 that enhanced transduction ability within retinal tissues [40]. THR peptide directly interacted with AAV8 and promoted the BBB penetration of AAV8 in the brain [19]. PhP.B peptide enhanced BBB transduction 40-fold in the AAV9 serotype [41]. CPPs, such as TAT-HA2 or LAH4, incubated with AAV6 increased transduction efficiency in Jurkat T cells 10-fold [27]. Synthetic CPPs such as Tat-Y1068 improved AAV2 transduction efficiency in fibroblast and epithelial cells in a dose-dependent manner [42]. CPPs also have useful characteristics that reduce the immunogenicity of AAVs. For example, an anti-inflammatory CPP, KAFAK, penetrated the BBB and reduced the production of proinflammatory cytokines [43]. Another example of a prion protein-derived CPP (Prp peptide) has been shown to exhibit anti-amyloid properties and re-direct pathways to reduce cell-toxic molecular structures in vivo [44]. Together, previous studies suggest that CPPs have potential in addressing the flaws (low efficiency, low penetration efficiency of BBB, immune response, and toxicities) of AAV delivery.

## 2. Cell-Penetrating Penta-Peptides (CPP5s)

### 2.1. Background History of the Discovery and Invention of CPP5s

Among various CPPs, recent studies showed that Cell-Penetrating Penta-Peptides (CPP5s) significantly enhanced BBB/BRB penetration of AAVs without problematic inflammatory reaction in rodents and primate models [23,45,46]. CPP5s are a group of peptides comprising five amino acids originally designed from the Bax-binding domains of Ku70 of various species (human, mouse, rat, chicken, frog, etc.) and their negative control peptides, which retain CPP activity without Bax-inhibiting activity (Table 1) [1,2,47].

Bax is a proapoptotic member of the Bcl-2 family of proteins that is an evolutionary conserved protein regulating mitochondria-dependent cell death [48]. Ku70 was identified as an inhibitor of Bax-induced cell death through a yeast-based functional screening study [1,2]. Previously, Ku70 was known as an evolutionarily conserved protein functioning in DNA double-strand break repair mechanisms [49]. Several studies showed that Ku70 inhibits Bax-induced cell death in mammalian cells by inhibiting the conformational change and mitochondrial translocation of Bax [1,3,47,50,51,52]. This Bax inhibition activity has been shown to be modified by post-translational modifications of Ku70 (ubiquitinylation and acetylation) [50,51,53,54]. Furthermore, Bax gene knockout extended the lifespan of Ku70 knockout mice exhibiting accelerated aging [55,56], supporting the physiological significance of Ku70-depedent Bax inhibition. To determine the molecular mechanism of Ku70-mediatred Bax inhibition, the Bax binding domain of Ku70 has been identified, and penta-peptides mimicking the Bax binding domains of Ku70 were designed [1,57]. These peptides were named Bax-Inhibiting Peptides (BIPs). Although BIPs showed cell death inhibition activity in certain conditions, they require relatively a high concentration (more than 200 μM) for cell death inhibition [3]. Due to this weak anti-cell death activity, the potential of BIP as a cell death inhibiting therapeutic is not high. However, BIPs did not show cytotoxicity even at 1.6 mM concentration suggesting that BIPs have a favorable “non-toxic” character as CPPs to deliver cargo molecules [3].

Importantly, negative control peptides of BIP were developed that have CPP activity but not Bax-inhibiting activity [3]. To design negative control peptides, the amino acid sequence was flipped or randomized, and their cell entry activities were examined. Notably, KLPVM (one of the mutated peptides that do not inhibit Bax) showed the best cell-penetrating activity among the tested CPP5s [3]. After realizing the potential of the negative control pentapeptides as CPPs, additional pentapeptides were designed based on these peptides. For example, based on KLPVM, modified pentapeptides such as KLGVM were designed. KLGVM was recently rediscovered by Dr. Gao’s team, who showed that KLGVM can enhance the gene delivery activity in the retina as explained later [45].

### 2.2. Mechanism of Cell Penetration of CPP5s

The frequently used CPP for cargo delivery (e.g., peptide) is the Tat-peptide comprising 11 amino acids (sequence “YGRKKRRQRR”) that contain eight positively charged amino acids (6Rs and 2 Ks) [28]. Importantly, several other CPPs have been found to contain a high proportion of cationic amino acids (K and R), and a simple poly-Arginine (R) peptide, such as R8, has been shown to possess CPP activity [28,58]. These cationic CPPs (including Tat-peptide) are hypothesized to interact with negatively charged cell surface molecules, such as proteoglycans and other cell surface molecules containing negatively charged portions. This interaction triggers internalization through clathrin-mediated or caveolae-mediated endocytosis [34,37,59]. The mechanism of how endocytosed peptides (and the conjugated cargo molecules) exit from the endosome to enter the cytosol and nucleus is not completely understood, but the acidification-dependent dissociation of the cationic peptide from the receptors and/or vesicle membrane in the endocytosed vesicle has been proposed as the potential mechanism for the cytosol entry of the CPP [60,61]. In the case of other CPPs (non-cationic CPPs), another hypothetical model has been proposed in which CPPs may also cause direct translocation by inducing pores in the lipid bilayer or by vesicle budding, though each CPP may use its own specific mechanisms for cell entry and cargo delivery [60,61].

As for CPP5s, measurements of isotope-labeled peptides inside of cells using mass spectrometric analysis demonstrated that CPP5s are likely utilizing a receptor-mediated cell entry mechanism [3]. Although the receptor itself has not yet been identified, the efficiency of the cell-penetrating activities of CPP5s is limited by the availability of the putative CPP5 receptor [3]. To utilize CPP5s to improve AAV- or nanoparticle-mediated drug delivery, the identification of the receptor (or mediator) will be necessary to further improve the drug delivery technologies [3].

The cell-penetrating activity of CPP5s was also observed even in specific cells lacking proteoglycan (a negatively charged molecule on the plasma membrane) [3,62]. Unlike cationic CPPs, most of CPP5s consist of four non-polar or uncharged amino acids and only one positively charged amino acid (e.g., K or E) (Table 1). Therefore, CPP5s are unlikely to interact with negatively charged cell surface molecules such as proteoglycans [63,64]. CPP5s also exhibited cell-penetrating activity in Jurkat cells, which has very low caveolin-mediated endolytic activity [65].

In comparison with polycationic CPPs (e.g., Tat), CPP5 cell entry activity was relatively low when the activity was compared by using cell entry activities in cultured cells [3]. For example, the cell entry activity of CPP5 was approximately 10 times lower than that of the Tat peptide [3]. Although CPP5s have lower cell entry activity than the Tat peptide, CPP5s have much less cytotoxicity than the Tat peptide [3]. Tat and poly-arginine peptides are known to show cytotoxicity when they are used at high doses, such as 100 μM or higher, likely due to their cationic properties [3]. On the other hand, CPP5s were not toxic even at 1.6 mM in cell culture [3]. Although the Tat peptide is frequently used for bioactive peptide delivery, especially in the neuroscience field, the Tat peptide is a highly cationic peptide, and it can non-specifically bind negatively charged surface molecules, including proteins, nucleotides, and lipids. Due to this chemical character of the Tat peptide, the use of the Tat peptide requires caution about the specificity and toxicity. On the other hand, CPP5s are not highly charged peptides as explained earlier in Section 2.2, and they do not randomly neutralize or stick to negatively charged molecules in the cell [3]. These unique features of CPP5s require further research to identify the primary receptor mediating mechanism for cell entry.

### 2.3. Hypothetical Mechanism of BBB Penetration by CPP5 (Figure 1)

At present, the mechanism by which CPP5 can improve AAVs’ BBB penetration remains enigmatic. As explained earlier, CPP5 contains only one cationic amino acid whereas Tat contains eight cationic amino acids among 11 amino acids [28]. CPP5 has hydrophobic (lipophilic) amino acids and it has higher affinity to membrane lipids than Tat and other cationic CPPs. This unique character of CPP5 may help AAVs to penetrate the BBB. For instance, after the cell entry of CPP5-conjugated AAVs, the lipophilic part of CPP5 may increase the chance of the association with the membrane of exocytotic vesicles in the endothelial cells. The putative CPP5 receptor may be present on the membranes of both endocytosis and exocytosis. In the case of Tat, after the cell entry, the highly positively charged Tat can be strongly attracted by negatively charged molecules in the cell, including DNA, and this condition is likely to decrease the ability of Tat-conjugated AAVs to exit from the cell. In fact, it is very well known that the Tat-conjugated peptide accumulates in the nucleus due to its high affinity to DNA [66]. On the other hand, CPP5 has been shown to distribute mainly in the cytosol. In addition, another logically possible mechanism is the penetration through the intercellular gap. Since recent studies showed that CPP5 did not increase BBB leakiness [23,67], CPP5 does not allow AAVs to penetrate the BBB by creating loose holes in the intercellular gap. If this mechanism exists, CPP5-conjugated AAVs may use an unrecognized mechanism to traffic AAV particles without increasing BBB leakiness. Further investigation is warranted to uncover the mechanism of BBB penetration by CPP5 to develop safer and more efficient technologies to deliver therapeutic molecules and AAVs through the BBB.

**Figure 1:**
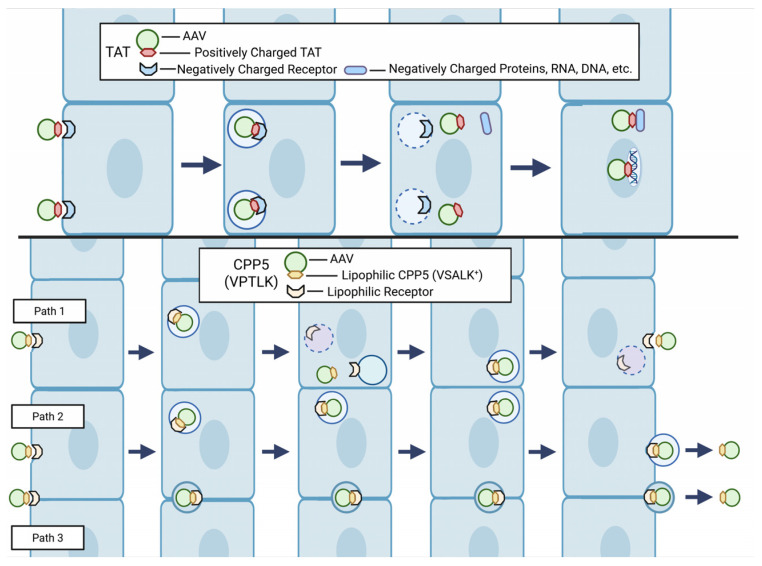
Hypothetical mechanism of BBB penetration by CPP5-conjugated AAVs. (**Above**) AAVs conjugated with a frequently used CPP, such as Tat, can enter cells through the CPP’s high affinity to negatively charged molecules, including DNA. However, this strong interaction may make it difficult for CPP-conjugated AAVs to exit from the cells forming the BBB. (**Below**) CPP5 contains hydrophobic (lipophilic) and uncharged non-polar amino acids. These amino acids that have affinity to the cellular membrane may allow CPP5-conjugated AAV to be transferred to the exocytotic vesicle membranes (Path1). Alternatively, CPP5 receptor-like molecules may exist in the membranes associated with exocytosis (Path2). Another speculative mechanism is the penetration of the cell–cell gap between cells by previously unknown mechanisms (Path3). Created in BioRender. Diener, A. (2026) https://BioRender.com/mvnetvo, accessed on 4 October 2025.

### 2.4. Potential Mechanisms of How CPP5 Can Evade Immune Reaction (Figure 2)

Recent studies have confirmed that CPP5-conjugated AAVs did not elicit a significant immune response [23]. The mechanism of how CPP5-conjugated AAVs can evade the immune system is not understood. Since CPP5 has a hydrophobic portion, lipophilic molecules (small lipids) in the blood and interstitial space may weakly associate with the CPP5, which may result in masking CPP5 from immune detection. The original CPP5s were designed from Ku70 protein that is expressed at high levels in immune cells, especially lymphocytes; Ku70 is an essential protein in the non-homologous end joining double-strand DNA break repair machinery which is required for immunoglobulin gene rearrangement in lymphocytes [49]. Therefore, the immune system may have relatively weak sensitivity against peptide sequences originating from Ku70 (e.g., CPP21 used for AAV [23]).

**Figure 2:**
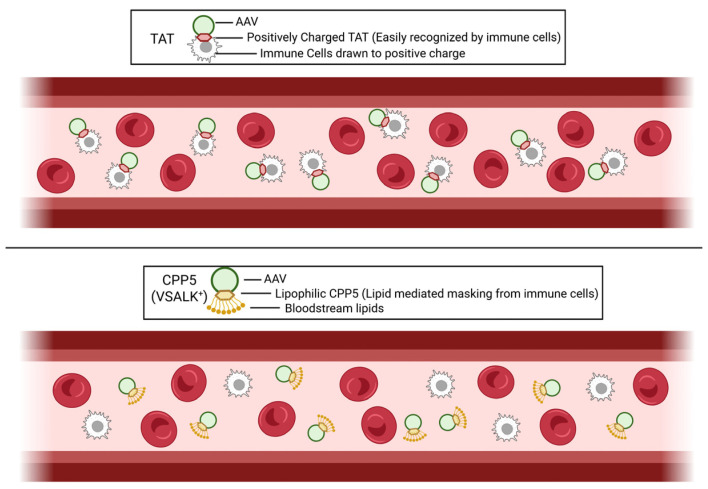
Hypothetical mechanism of immune evasion of CPP5-conjugated AAV. (**Above**) In the case of Tat-conjugated AAVs, the positively charged Tat can be easily detected by immune cells. (**Below**) Due to the presence of lipophilic amino acids in CPP5, CPP5-conjugated AAVs may be masked by lipids in the blood that can decrease the detection by immune cells. Created in BioRender. Diener, A. (2026) https://BioRender.com/0yvzcdt, accessed on 4 October 2025.

## 3. CPP5s Enhance AAV9 Delivery in the Brain of Mice and Non-Human Primates (Figure 3)

In 2022, Dr. Bei’s laboratory demonstrated utilization of VSALK, a CPP5, can improve the gene delivery and BBB penetration of AAV9 [23]. They tested 14 CPP sequences and found two CPP5s, VPALR and VSALK, were the most optimal at improving BBB penetration. Since these CPP5s were originally designed from Ku70 [1,2], the research team further tested TVSALK based on the original Ku70 amino acid sequence. The team found that TVSALK showed further improved gene transduction activity in comparison with VSALK, and they named this sequence (TVSALK) as CPP16. This peptide insert was further modified by adding F between L and K creating the variant AAV.CPP.21 (CPP.21 has TVSLFK sequence). Intravenous injections of AAV.CPP.16 and AAV.CPP.21 showed an increasing gene transduction efficiency of Red Fluorescent Protein (RFP) of up to 249-fold in comparison with the control AAV in four strains of adult mice brain cells. Consistently, AAV.CPP16 and AAV.CPP21 showed higher brain transduction than unmodified AAV9 intravenous injection, with no significant difference between AAV.CPP.16 and 21 [23].

**Figure 3:**
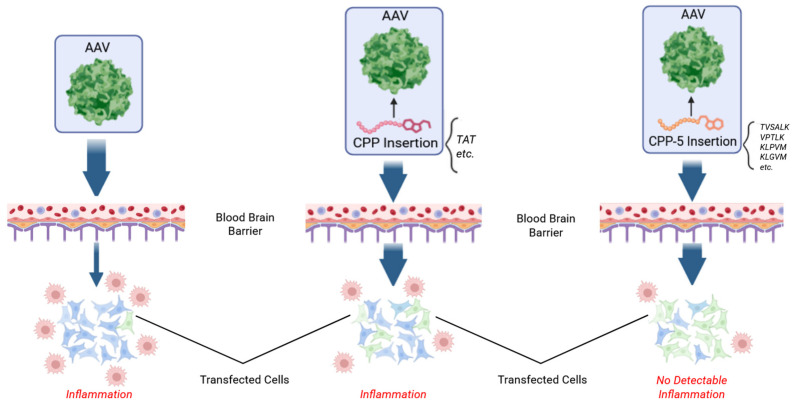
Utilization of CPP5s increases the penetration of the blood–brain barrier (BBB) and minimizes immune reactions [23]. AAV: Adeno-associated virus; CPP: Cell-Penetrating Peptide; CPP5: Cell-Penetrating Penta-Peptide. Created in BioRender. Diener, A. (2025) https://BioRender.com/q17wmho, accessed on 4 October 2025.

Furthermore, the authors confirmed that CPP5-containing AAV9 penetrated the BBB in primate brains. By using CPP.16 and 21, the efficiency of AAV9-mediated gene delivery to the brain cortex increased to 7.0-fold and 1.9-fold compared to the AAV9 without CPP, respectively. An up to 13.2-fold increase for AAV.CPP.16 and up to 20-fold increase in AAV.CPP.21 transduction efficiency in other brain regions were also established. At high doses, AAV.CPP.16 had greater transduction efficiency than AAV.CPP.21 in primates [23]. Previously it has been noted that higher doses of standard AAV vectors (without CPP.16 or CPP.21) carry the risk of leakage of the BBB, a pathological increase in membrane permeability. However, injection of dextran showed no leakage from the blood into the CNS and no difference in clearance between AAV.CPP.16 and AAV9 [23,67]. This suggests that CPP5-mediated AAV delivery does not cause harmful neuroinflammation or destruction of the BBB in primates, as typically seen with AAV vectors alone.

## 4. CPP5 Containing CPP.16 Enhances AAV Delivery in the Respiratory Tract

AAV.CPP.16 was further evaluated for transduction efficiency in airway and lung cell tissues [68]. In cultured human nasal epithelial cells, AAV.CPP.16 was 3.0-fold and 6.6-fold better than AAV6 and AAV9 at delivering a Green Fluorescence Protein (GFP) reporter respectively. Intranasal administration of AAV.CPP.16 into the mouse nasal cavity, trachea, and lung all showed similar increases in GFP-derived fluorescence in comparison with wild-type AAV9 by 2.7-fold and AAV6 by 1.8-fold. Furthermore, AAV.CPP.16 penetrated deeper into lung and respiratory tissue, all with no-to-little transduction into other peripheral tissue such as the brain, retina, or muscle. In the nasal cavity, AAV.CPP.16 transduced 42.7% of goblet cells, while transduction of AAV6 was only 3.0% and AAV9 was 7.8%. In the trachea, AAV.CPP.16 transduced 71.0% of club cells, compared to 29.6% for AAV6 and 20.2% for AAV9. In the lung, AAV.CPP.16 transduced 13.6% of ciliated cells, compared to 2.8% for AAV6 and 1.9% for AAV9. In non-human primates, intranasal delivery of CPP.16 improved AAV9-mediated GFP gene delivery into the respiratory tract up to 10.5-fold compared to AAVs lacking CPP. Notably, no AAV.CPP.16 expression was found in peripheral tissues and no abnormal weight loss or adverse effects were noted in mice or non-human primates [68].

In addition to reporter genes, AAV.CPP.16 can also deliver decoy receptors of TGF-β1 to treat pulmonary fibrosis within mice. This decoy receptor induced by AAV.CPP.16 significantly reduced the protein levels of TGF-β1 and VEGFα compared to non-treated mice. Furthermore, there were significantly reduced levels of mRNA for fibrogenic genes and inflammatory cytokines. This resulted in reduced expression of extracellular matrix proteins, decreased inflammatory cytokines, and reduced fibroblast-associated proteins in mouse lungs after CPP-enhanced trap treatment [68].

CRISPR RNA endonuclease targeting viral RNA in SARS-CoV-2 was also modified with AAV.CPP.16 to measure prophylactic effectiveness. In this mouse pseudovirus model, CasRx-gRNA.Niran and CasRx-gRNA.Interface (RNA targeting Cas nucleases) were packaged into AAV.CPP.16, and Rdrp (RNA-dependent RNA polymerase) transcription of the pseudovirus was reduced by 50% and 60% respectively. Despite the pseudovirus inducing expression 50–300-fold in mouse respiratory tissues, AAV.CPP.16-mediated treatment caused the near-complete block of Rdrp in the nostrils and was reduced by 70% in the trachea and 67% in the lung [68]. These results suggest that CPP-integrated AAVs may be able to improve the efficiency of CRISPR/Cas9-mediated gene therapy in the future.

## 5. CPP5 Enhances AAV2 Delivery in the Retina (Figure 4)

In another recent study, AAV2s containing CPP5s were injected intravitreally to evaluate effects on gene delivery to mice retinal cells [46]. He et al. found that AAV2.CPP.21 had the highest level of pan-retinal reporter gene delivery (the GFP gene was used as a reporter), with significant GFP expression in the outer nuclear layer (photoreceptor) and inner nuclear layer (bipolar cells). Non-modified AAV2 was found to primarily transduce cells in the retinal ganglion cell (RGC) layer, whereas AAV2.CPP.16 mediated fluorescence expression in deeper retinal cells. Notably, AAV2.CPP.21 was found to have the deepest penetration extending to the outermost RPE layer without noticeable retinal toxicity or transgene leakage in peripheral organs. Overall, CPP-integrated recombinant AAV2 showed an improved efficiency of AAV2 delivery and targeting of the retina.

**Figure 4:**
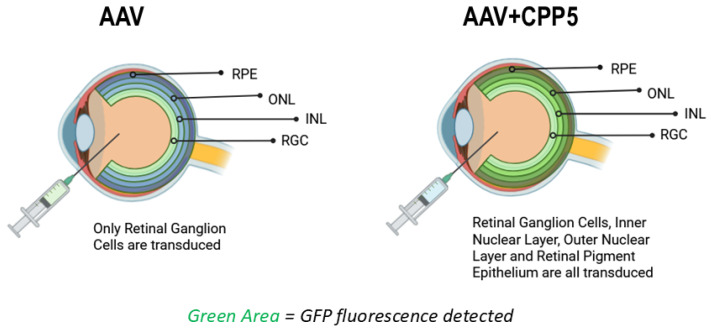
Utilization of CPP5 increases the penetration of the blood–retina barrier (BRB) [57,65]. AAV: Adeno-associated virus; CPP5: Cell-Penetrating Penta-Peptide; RPE: Retinal Pigment Epithelial Cells; ONL: Outer nuclear layer (Photoreceptor); INL: Inner nuclear layer (Bi-polar cells); RGC: Retinal ganglion cells. Created in BioRender. Diener, A. (2025) https://BioRender.com/glu4j9i, accessed on 4 October 2025.

In *rd1* mice (*Pde6b* gene mutation mouse model of retinitis pigmentosa), He et al. observed that AAV2.CPP.21 delivery of the *Pde6b* gene rescued pupillary light reflex behavior and restored thickness of the retina when compared to AAV2 [46]. This was further confirmed by RNA sequencing and PCR, showing dramatically elevated gene expression of *Pde6b* and phototransduction in mice treated with AAV2.CPP.21 when compared to AAV2. The AAV2.CPP.21-treated group had a stronger visual perception ability and photo transduction genes, indicating that AAV.CPP.21-mediated gene delivery effectively attenuated photoreceptor degeneration in *rd1* mice [46].

## 6. Mechanisms of How CPP.16 and CPP.21 Help AAV Gene Delivery

The precise molecular mechanism of how CPP.16 and CPP.21 enhance gene delivery of AAVs is not fully understood. Since CPP.16 (TVSALK) is similar to the BIP designed from the Bax binding domain of Ku70, there is a possibility that CPP.16 increased the survival of host cells by inhibiting Bax-mediated cell death. However, this possibility is low. BIP requires at least 200 μM concentration to achieve cell death inhibition [3], and the extracellular concentration of AAV.CPP16 in the target tissues (e.g., the brain, lung, and retina) would have been much lower than 200 μM in the experimental condition testing gene delivery in animal models. Notably, Dr. Bei’s team reported that they observed that AAV.CPP16 did not decrease Bax expression levels in HEK293T. Based on this result, they hypothesized that AAV.CPP16 does not function as a Bax inhibitor. Moreover, it was initially shown that Ku70 directly binds and inhibits Bax without changing Bax expression levels [1,3,47,50,51,52]. The mechanism of Bax inhibition by Ku70 is to inhibit the Bax conformational change, but Ku70 does not alter Bax expression levels [3,47,50]. Furthermore, Ku70 has been reported to be expressed on the cell surface in addition to the nucleus and cytosol, and Ku70 functions as the receptor for extracellular molecules including parasitic organisms [69]. CPP.16 and CPP.21 may thus bind to Ku70-interacting cell surface molecules to facilitate entry into the host cell. Table 2 summarizes the CPP5 utilized for AAV gene delivery, and the cell types infected.

## 7. KLGVM, a CPP5, Enhances AAV2 Delivery and Reduces Immunogenicity in the Retina (Figure 2)

Wang et al. reported that three versions of CPP5s (VPTLK, KLPVM, and KLGVM), significantly improved the gene transduction of AAV2 in the retina [45]. It has been shown that the extracellular matrix of the retina has heparan sulfate proteoglycans which bind (and trap) AAV2, and this interaction prevents the entry of AAV2 into the retinal cells [45]. Unlike cationic CPPs, CPP5s do not utilize negatively charged glycosaminoglycans as the receptor for cell entry, as explained in Section 2.2. After screening 1070 CPPs, Wang et al. identified KLGVM as the best CPP, which allows AAVs to escape from the heparan sulfate proteoglycan trap. Other CPP5s, such as VPTLK and KLPVM also enhanced enrichment in the retina, but KLGVM was the most effective. They named the KLGVM-integrated AAV2 as AAV2.CPP1 retina [45]. The CPP5 sequence (KLGVM) was inserted within the VR-VIII region of the AAV Capsid, potentially interrupting the R585 and R588 binding motif of heparin sulfate proteoglycans in the retina [45].

Following intravitreal injection in mice, AAV2.CPP1 transduced the reporter GFP gene in photoreceptors 2.5-fold more than ssAAV2.7m8.CB6.GFP (an engineered AAV with CPP insertion), a previously optimized AAV vector for retinal cell transduction. The transduction of AAV2.CPP1 was observed to be at a higher proportion in Müller cells, and present in the outer nuclear layer (the photoreceptor layer) to the retinal ganglion cell layer [45].

Similarly to AAV.CPP1, AAV2.7m8 was developed as an AAV2 variant that has a low affinity to heparan sulfate proteoglycan [70]. Although AAV2.7m8 has an increased efficiency in gene transduction to the target tissue, AAV2.7m8 also increased immune response of the host tissue in comparison with the original AAV2. For example, AAV2.7m8 induced microglial cell migration into the inner plexiform layer more than the original AAV2. AAV2.CPP1, however, did not have an increased immune response in treated retinas. In fact, the RNA levels of inflammatory cytokines such as IL-6 were significantly lower in AAV2.CPP1 when compared to AAV2.7m8 in the retina [45]. Compared with AAV2.7m8, KLGVM-engineered AAV2 (AAV2.CPP1) exhibited increased transduction efficacy and reduced inflammatory response.

## 8. Other Applications of CPP5s for Therapeutic Protein Delivery into Various Cell Types in Animal Models

In addition to AAV-based technology, CPP5s as well as other CPPs have been used to deliver bioactive molecules into the target cells both in vitro and in vivo [71,72]. In 2024, Hołubowicz et al. reported that CPPs were able to deliver Cre-DNA recombinase protein into the retinal photoreceptor in mice when the CPP-conjugated Cre protein was administrated by intraocular injection [73]. In this study, three representative CPPs, including CPP5, Tat, and ANTP, were fused to the Cre-recombinase protein, and all the tested CPPs effectively delivered the Cre protein into the retina. The intracellular delivery of Cre was validated by Cre-recombinase-dependent GFP expression in the retina. Although CPP5 was able to deliver the Cre-protein in the retinal cells in vivo, the study reported that the Cre-protein delivery activity was not detectable in vitro (cell culture system). The negative result may be due to the low concentration of CPP5–Cre protein. In this study, only 1 μM concentration of CPP5–Cre was used [73]. In previous studies, the cargo delivery activity of CPP5 became clearly detectable from 10 μM, but the activity was not detectable at 1 μM in cell culture. This may explain the negative result from Hołubowicz’s report, and is consistent with previous studies. Notably, Kang et al. (2021) reported that CPP5–Cre delivered the Cre protein into the primary cultured porcine fibroblast and CPP5–Cre (4 μM concentration was used) achieved 90% efficiency of Cre-dependent gene recombination [74]. In this study by Kang et al., CPP5 showed the best gene recombination induction among the three CPPs examined (CPP5, Tat, and R9) [74].

There are other successful applications of CPP5s for protein transduction both in vivo and in vitro. The examples of these applications are: (1) In mice, VPALR, one of the CPP5s, was conjugated to sulpiride (atypical antipsychotic medication in a benzamide class) and CPP5 increased the drug bioavailability, half-life, and increased transport across the BBB to enhance antidepressive effects [75]. (2) VPTLK enhanced mini-chaperone activity of the peptides derived from alphaA-crystallin protein (amino acids 70–88) and prevented β-amyloid-induced cell death [76]. (3) The CPP5 conjugation to the AL3810 liposome attenuated the immune response induced by liposome and improved the delivery of an anti-tumorigenic compound targeting glioma [77]. (4) QLPVM was found to allow transferrin-coated liposome to penetrate the BBB in a mouse model and QLPVM-conjugated liposome was able to deliver doxorubicin (an anti-cancer drug) across the BBB [78].

## 9. Potential Problems and Future Direction

AAVs have been considered a relatively safe vector for gene therapy [79,80]. In ophthalmology, AAV-mediated gene delivery for a recessive genetic disease (PDE6b mutation-induced retinitis pigmentosa) has been approved by the FDA [81]. On the other hand, AAV-mediated gene therapy for Duchenne muscular dystrophy is now confronting serious risks due to the three fatal incidents of patients with an immune reaction against the AAV therapy [17,82,83]. Fundamentally, all AAV delivery systems (including CPP5-integrated AAVs) share similar inherent limitations regarding the possibility of triggering cellular immune responses. While the peripheral toxicity of CPP5-integrated AAVs was observed to be low in preclinical models, further careful studies (including reproducibility studies in multiple animal models, especially in non-human primates) will be required before considering human studies.

## Figures and Tables

**Table 1:** List of CPP5s. Their cell penetration activity was tested and confirmed as previously reported [1,2,3,4]. CPP5: Cell-Penetrating Penta-Peptide; Ku70: DNA binding protein; Bax: Pro-apoptotic protein.

Sequence	Origin and Background History
VPMLK	Bax binding domain of human, monkey and dog Ku70
VPTLK	Bax binding domain of mouse Ku70
VPALR	Putative Bax binding domain of rat Ku70
VSALK	Putative Bax binding domain of chicken Ku70
PMLKE	Bax binding domain of human, monkey and dog Ku70
VPALK	Putative Bax binding domain of Cattle and African clawed frog Ku70
VSLKK	Artificially designed CPP5
VSGKK	Artificially designed CPP5
KLPVM	Artificially designed CPP5
IPMIK	Artificially designed CPP5
KLGVM	Artificially designed CPP5
KLPVT	Artificially designed CPP5
VPMIK	Artificially designed CPP5
IPALK	Artificially designed CPP5
IPMLK	Artificially designed CPP5
VPTLQ	Artificially designed CPP5
QLPVM	Artificially designed CPP5
ELPVM	Artificially designed CPP5
VPTLE	Artificially designed CPP5

Amino acids with nonpolar side chains: A, G, C, V, P, L, M, F, W. Amino acids with uncharged polar side chains are listed in blue: N, Q, S, T, Y. Amino acids with charged polar side chains are listed in red: R, K, E, H, D.

**Table 2:** List of utilized CPP5 and CPP5 derivatives that improved transduction efficacy by infected species and cell type. BBB: Blood–brain barrier, RGC: Retinal Ganglion Cells, ONL: Outer nuclear layer, INL: Inner nuclear layer, RPE: Retinal Pigment Epithelium.

Sequence	Species	Cell Type
TVSALK (CPP.16)	Human	Astrocytes^23^, hCMEC/D3^23^ (BBB cell line), HEK293T^23^ (embryonic kidney), RPMI 1680 cells^67^ (nasal epithelium)
	Primate	Nerons^23^, astrocytes^23^, oligodendrocytes^23^, spinal motor neurons^23^, goblet cells^67^, club cells^67^, bronchial ciliated cells^67^, alveolar type 1 cells^67^
	Mouse	Nerons^23^, astrocytes^23^, oligodendrocytes^23^, goblet cells^67^, club cells^67^, bronchial ciliated cells^67^, alveolar type 1 cells^67^, RGC^68^, ONL^68^, INL^68^, RPE^68^
TVSLFK (CPP.21)	Human	Astrocytes^23^, hCMEC/D3^23^, HEK293T^23^
	Primate	Nerons^23^, astrocytes^23^, oligodendrocytes^23^
	Mouse	Brain^23^, GL261 glioma^23^, RGC^68^, ONL^68^, INL^68^, RPE^68^
KLGVM (CPP1)	Human	HeLa Cells^57^ (Cervical)
	Mouse	RGC^57^, Müller Cells^57^, Photoreceptors^57^
KLPVM	Human	HeLa Cells^57^ (Cervical)
	Mouse	RGC^57^, Müller Cells^57^, Photoreceptors^57^
VPTLK	Human	HeLa^57^ (Cervical)
	Mouse	RGC^57^, Müller Cells^57^, Photoreceptors^57^

## Data Availability

No new data were created or analyzed in this study. Data sharing is not applicable to this article.

## Abbreviations

The following abbreviations are used in this manuscript:

CPP5	Cell-penetrating penta-peptide
AAV	Adeno-associated virus
BBB	Blood–brain barrier
BRB	Blood–retina barrier
CPP	Cell-penetrating peptide
BIP	Bax-inhibiting peptide
GFP	Green fluorescent protein
RPE	Retinal pigment epithelium
RGC	Retinal ganglion cell
Rdrp	RNA-dependent RNA polymerase
